# The effect of geographic origin and destination on congenital heart disease outcomes: a retrospective cohort study

**DOI:** 10.1186/s12872-023-03037-w

**Published:** 2023-02-22

**Authors:** Spencer M. Millen, Cara H. Olsen, Ryan P. Flanagan, John S. Scott, Craig P. Dobson

**Affiliations:** 1 Walter Reed National Military Medical Center, Department of Pediatrics, Uniformed Services University of the Health Sciences, Bethesda, MD USA; 2 Department of Preventive Medicine and Biostatistics, Uniformed Services University of the Health Sciences, Bethesda, MD USA; 3 Tripler Army Medical Center, Honolulu, HI USA; 4 Veterans’ Health Administration, Washington, DC USA

**Keywords:** Congenital heart disease (CHD), Distance, Rurality, Rural, Urban, Society of thoracic surgery (STS)

## Abstract

**Background:**

Congenital heart disease (CHD) is a common and significant birth defect, frequently requiring surgical intervention. For beneficiaries of the Department of Defense, a new diagnosis of CHD may occur while living at rural duty stations. Choice of tertiary care center becomes a function of geography, referring provider recommendations, and patient preference.

**Methods:**

Using billing data from the Military Health System over a 5-year period, outcomes for beneficiaries age < 10 years undergoing CHD surgery were compared by patient origin (rural versus urban residence) and the distance to treatment (patient’s home and the treating tertiary care center). These beneficiaries include children of active duty, activated reserves, and federally activated National Guard service members. Analysis of the outcomes were adjusted for procedure complexity risk. Treatment centers were further stratified by annual case volume and whether they publicly reported results in the society of thoracic surgery (STS) outcomes database.

**Results:**

While increasing distance was associated with the cost of admission, there was no associated risk of inpatient mortality, one year mortality, or increased length of stay. Likewise, rural origination was not significantly associated with target outcomes. Patients traveled farther for STS-reporting centers (STS-pr), particularly high-volume centers. Such high-volume centers (> 50 high complexity cases annually) demonstrated decreased one year mortality, but increased cost and length of stay.

**Conclusions:**

Together, these findings contribute to the national conversation of rural community medicine versus regionalized subspecialty care; separation of patients between rural areas and more urban locations for initial CHD surgical care does not increase their mortality risk. In fact, traveling to high volume centers may have an associated mortality benefit.

## Background

The U.S. Department of Defense (DoD) provides health insurance for active duty service members and their dependents, including 1.9 million children [1]. This employment benefit makes the DoD one of the largest organizations funding children’s healthcare in the United States. As previously described, the military population is socioeconomically, demographically, and geographically diverse [2]. Congenital heart disease (CHD) surgeries for beneficiaries are funded by the DoD but exclusively performed at civilian centers. Such benefit is comprehensive, including continued income for the service member, travel costs, and lodging for the family if over a certain distance. Because of this comprehensive benefit, the DoD is the sole or primary payer for almost all CHD surgeries in this population. Cost and quality data are aggregated under TRICARE, the DoD’s single-payer insurance system, allowing comparison of outcomes across the beneficiary population.

The DoD seeks to provide consistent and reliable access to healthcare for the families of active duty service members regardless of their location. As mandated by Congress, duty station assignment of families with known complex healthcare needs must be coordinated with the Exceptional Family Members Program (EFMP) [3]. Dependents with chronic or life-threatening conditions enroll in EFMP to ensure that accepting duty stations have adequate healthcare and educational resources. However, as severe congenital heart defects may not be diagnosed until after birth, many pediatric beneficiaries are born or diagnosed within rural counties, where easy access to advanced pediatric surgical centers is limited.

Geographic separation of patients from tertiary care centers is a recognized barrier to healthcare [4–7]. However, given the unique care needs of patients with CHD, some argue for the consolidation of care in urban centers, at the expense of increasing distance for the nation’s rural population [8, 9]. There is precedence for this in other countries. Sweden, for example, made such a decision in 1993, centralizing CHD care into the two centers with the lowest surgical mortality. Following this decision, Sweden experienced a dramatic reduction in 30-day mortality, despite increased travel times for some patients [10].

Generally, studies have demonstrated decreasing mortality and cost with increasing surgical center volume [11–14]. This is particularly evident with high complexity procedures. Simulations have demonstrated a possible reduction in mortality with such a reorganization of healthcare delivery [8, 9]. If appropriately planned, regionalization has the additional advantage of standardizing referral networks for those publicly insured.

In an effort to advance the care of patients with CHD, the Society of Thoracic Surgeons (STS) manages an extensive outcomes database of congenital heart surgery hospitals. Participation within this database is voluntary, and membership now includes 113 congenital heart disease surgical centers, as of their 2019 report [15]. In the same year, there were at least 153 hospitals performing at least some CHD procedures [16]. While there has been an assumption that there would be improved outcomes in centers that publicly report their outcomes in the STS database, this has yet to be studied systematically.

This study seeks to characterize differences in healthcare outcomes based on rural versus urban residence, distance to the patient’s tertiary care center, and the reporting characteristics of that surgical center. While other studies have analyzed healthcare outcomes across rural–urban designations, complex regional insurance plans and varying coverage by state add significant complexity to regional comparisons. Active duty service members and their dependents are a nationally representative sample in terms of race and socioeconomic status, although importantly, they enjoy universal health care coverage. This distinction removes many confounding variables in the analysis of CHD outcomes and adds improved validity to comparison of care across state lines.

## Methods

### Study designs

The current study is a retrospective cohort design examining billing data from the Military Health System from 2011 to 2015. We analyzed all admissions for children (< 10 years) with CHD surgeries. All patients originated from within the United States or its territories. Subjects originating from foreign country locations were not included. In total, 1971 subjects were identified, accounting for 3384 admissions and 2430 procedures. Readmissions < 90 days after each surgery were determined. To adjust for differences in case complexity, an established algorithm, reported by Jenkins et al. [17] was used to risk-stratify the subjects using the validated Risk Adjustment for Congenital Heart Surgery (RACHS-1) surgical risk categories with ICD-9 codes.

Cost was defined as the billing charges for each admission. It was not delineated into the amount paid by TRICARE or outside insurance. As the duration of the study period was only five years, adjustment for inflation was not performed. The epoch notably included only ICD-9 diagnostic/procedure codes and did not include comparisons after the switch to ICD-10 in the United States.

Mortality data were queried from the Defense Enrollment Eligibility Reporting system (DEERS) and billing codes indicating that the patient died during admission. DEERS is regularly updated with data from the Social Security Administration and provides a reliable indicator of post-discharge mortality for military beneficiaries. Inpatient mortality reflected death during the first admission for a CHD procedure. One year mortality was defined as any death occurring within the first year following cardiac surgery, regardless of discharge date or readmissions.

Determination of facilities publicly reporting to the Society for Thoracic Surgery database (STS-pr) versus non-reporting (STS-nr) was based on those currently reporting as of December 2017. The STS reporting status was based on the STS reporting status in 2017 as the prior list was not publicly available. As multiple names may be used in billing for a given facility, billing zip codes were used to confirm the identity of hospitals that were not readily identifiable by billing name (for example, University Medical Associates).

To better address such high complexity care, center volume of STAT Category 4 and STAT Category 5 cases among reporting hospitals were used as the primary metric. Volume data for the closet available reporting period (2015–2018) was pulled from the STS Congenital Heart Surgery Public Reporting database [18]. Although only 1368 patients were treated in facilities identified as STS-pr by 2017, an additional 330 patients were treated in facilities for which high-complexity case volume information was available by 2019, and these patients are included in the analysis of case volume. Case volume information was not available for STS-nr facilities. In a 2020 policy review of CHD, authors describe a known relationship between center volume and patient outcomes [19]. However, this relationship is nuanced and likely reflects a larger picture of insurance programs, support systems, and individual clinician expertise [20]. In order to determine any undue risk in lowest volume centers, we selected the bottom quartile of annual high complexity cases for reference comparison. This focus on high complexity cases was determined important in future policy recommendations for shot- and long-term complex care management.

The initial patient residence zip code (postal code) was available for 1855 (94.1%) individuals within the cohort. Straight line distance between the patient’s home of record and each treatment hospital’s zip code was recorded in miles and available in the TRICARE dataset. To determine rurality, zip codes were cross-referenced with the rural–urban commuting area (RUCA) codes, available from the Economic Research Service of the US Department of Agriculture [21, 22]. The 1–10 scale ranks sub-county decennial census data from large urban centers “1” to rural areas “10”. For comparisons, we used the Urban core, Suburban, Large Rural Town and Small Rural Town scheme, described previously by the Washington State Department of Health [23]. Multivariate analysis was completed through comparison of Urban (Urban core and Suburban) versus Rural (Large and Small Rural Town). In this delineation, an urban core is a contiguous area of more than 50,000 people, while suburban areas are characterized by large commuting flows into the urbanized area. A large rural town is greater than 10,000 and less than 50,000 people. Finally, a small rural town has a population less than 10,000, generally with more than an hour travel time to the nearest city.

### Statistics

Key outcome variables were readmission within 90 days, cost of admission, inpatient mortality, and increased length of stay. Patient origin (rural versus urban residence) and the distance to treatment (patient’s home and the treating tertiary care center) were the primary predictors, with patient age, sex and RACHS-1 score and facility STS reporting status and high complexity case volume as potential confounders. Demographic differences (age, sex, rural–urban classification, CHD severity, and mortality) among beneficiaries were assessed via χ2. Distance was compared to RACHS-1 score, STS reporting status, and center volume using the Mann–Whitney U test. For the primary analyses, length of stay (LOS) and cost were analyzed with a Gamma-distributed generalized linear model for line of best fit. Multivariate logistic regression compared readmission within 90 days and inpatient mortality among independent variables. Analysis for readmission excluded patients who died during their primary admission. Hazard ratios (HR) for patient survival over a 1-year period were obtained using Cox multivariate logistic regression modeling. All analyses were completed in SPSS version 28.0.1.

## Results

### Descriptives

Of 1971 patients, 1047 were males (53.1%). 592 (30.0%) were < 1 month old, and 679 (34.4%) were between 1 month and 1 year at the time of their first procedure (Table 1). Patients with high complexity CHD, denoted as RACHS-1 scale 4–6, represented 325 (16.5%) of the population. 140 deaths were recorded within the study period. Higher RACHS-1 score was significantly associated with younger age and mortality.

**Table 1:** Demographics according to RACHS-1 categorization

	RACHS-1			
	Total (n = 1971)	0–3 (n = 1646)	4–6 (n = 325)	*p*
Mortality	140 (7.1)	87 (5.3)	53 (16.3)	< 0.001
*Age*				
< 1 months	592 (30.0)	321 (19.5)	271 (83.4)	< 0.001
1–12 months	679 (34.4)	646 (39.2)	33 (10.2)	
> 12 months	700 (35.5)	679 (41.3)	21 (6.5)	
*Sex*				
Male	1047 (53.1)	864 (52.5)	183 (56.3)	0.208
Female	924 (46.9)	782 (47.5)	142 (43.7)	
*RUCA*				
Urban	1642 (88.5)	1406 (89.0)	236 (85.5)	0.089
Rural	213 (11.5)	173 (11.0)	40 (14.5)	
Distance	193.8 (50.8)	184.0 (49.7)	249.5 (66.0)	0.017*

Count (%). Comparisons completed via χ2. *Mean (Median). Distance in miles. Significance for distance was calculated via Mann–Whitney U Test. RUCA, Rural–Urban Commuting Area. RACHS-1, Risk Adjustment for Congenital Heart Surgery

Of the cohort, 94.1% had available data on distance between home and treating hospital. Mean distance was 194 miles, however, median distance was only 51 miles, reflecting the presence of some extremely long distances. The longest distance between residence and treatment facility was 6185 miles, by a patient coming from the US territory of Guam to the continental United States. RACHS-1 severity was significantly associated with distance (Table 1).

The majority of patients 1501 (80.9%) lived within an urban core at the time of first admission, while 141 (7.6%) resided in a suburban location, 142 (7.7%) in a large rural town, and 71 (3.8%) in a small rural town. Under this designation system, all but one tertiary care center was within a code 1 area (urban core). The one children’s hospital located outside of an urban core was within a code 2 area (metropolitan, with high commuting into an urban core).

Analyzing for the first procedure, 1368 (69%) were at an STS-pr facility and 606 (31%) at an STS-nr facility (Table 2). Overall, 23% of procedures were followed by readmission within 90 days. Procedures were performed in 47 states and the District of Columbia. Seven centers accounted for 622 (32%) of the 1971 admissions. Those were Rady Children’s Hospital (*n* = 164), Emory (*n* = 93), Seattle Children’s Hospital (*n* = 91), Children’s National Medical Center in DC (*n* = 80), Children’s Hospital of Philadelphia (*n* = 67), Children’s Hospital of Colorado (*n* = 65), and Duke University Hospital (*n* = 62).

**Table 2:** Demographics according to STS reporting status and center volume

	STS center reporting status			Annual high-complexity case volume		
	STS-nr (n = 603)	STS-pr (n = 1368)	*p*	< 50 (n = 400)	> 50 (n = 1298)	*p*
RACHS-1			0.735			0.510
0–3	501 (83.1)	1145 (83.7)		339 (84.8)	1082 (83.4)	
4–6	102 (16.9)	223 (16.3)		61 (15.3)	216 (16.6)	
Age			0.629			0.003
< 1 month	178 (29.5)	414 (30.3)		95 (23.8)	420 (32.4)	
1 month–1 year	217 (36.0)	462 (33.8)		140 (35.0)	432 (33.3)	
> 1 year	208 (34.5)	492 (36.0)		165 (41.3)	446 (34.4)	
Sex			0.343			0.008
Male	330 (54.7)	717 (52.4)		234 (58.5)	661 (50.9)	
Female	273 (45.3)	651 (47.6)		166 (41.5)	637 (49.1)	
RUCA			0.326			0.729
Urban	509 (89.6)	1133 (88.0)		340 (88.8)	1068 (88.1)	
Rural	59 (10.4)	154 (12.0)		43 (11.2)	114 (11.9)	
Distance*	119.5 (48.4)	230.9 (52.4)	0.008	193.2 (45.8)	275.0 (62.9)	< 0.001

Count (%). *Comparisons completed via χ2 *Mean (Median), Distance in miles. RUCA, Rural–Urban Commuting Area. RACHS-1, Risk Adjustment for Congenital Heart Surgery. STS-pr, Society of Thoracic Surgeons database publicly reporting. STS-nr, Society of Thoracic Surgeons database not publicly reporting. Center volume represents annual volume of high complexity procedures (STAT Category 4 and STAT Category 5)

Patients initially treated at STS-pr facilities lived farther from their surgical center as compared to patients treated at non-reporting centers (median distance 231 miles to 120 miles, respectively). This difference was more pronounced for centers that performed > 50 high risk cases annually (Table 2). Patients at low volume centers were more likely to be older and male.

### Distance and urban–rural classification

Rural residence was not associated with any related outcome variables, to include one year mortality, in-hospital mortality, LOS, cost, and readmission (Table 3). With the same covariates, straight-line distance was positively associated with cost (*p* = 0.001 β = 1.198 (95% CI 1.076–1.334)). Estimated means of billed cost between patients traveling > 150 miles and < 15 miles was $408,221 and $340,714 respectively.

**Table 3:** Outcomes by distance, rural designation, STS reporting status and center volume

	Cost				Length of stay				Readmission within 90 days				Inpatient mortality			
	Exp (B)	95% CI		Sig	Exp (B)	95% CI		Sig	Exp (B)	95% CI		Sig	Exp (B)	95% CI		Sig
*Distance (Miles)*																
< 15	Reference				Reference				Reference				Reference			
15–50	0.998	0.899	1.108	0.969	0.972	0.864	1.093	0.634	1.239	0.864	1.777	0.244	0.941	0.415	2.136	0.885
50–150	0.935	0.843	1.038	0.207	1.059	0.942	1.191	0.339	1.316	0.955	1.815	0.094	1.422	0.691	2.929	0.339
> 150	1.198	1.076	1.334	0.001	1.115	0.987	1.259	0.079	0.916	0.662	1.268	0.598	0.766	0.371	1.580	0.470
*Rural–urban commuting area*																
Urban	Reference				Reference				Reference				Reference			
Rural	0.932	0.831	1.045	0.229	1.015	0.892	1.156	0.817	1.282	0.898	1.828	0.171	0.408	0.126	1.323	0.135
*STS reporting status*																
Non-reporting	Reference				Reference				Reference				Reference			
Reporting	0.929	0.859	1.004	0.064	1.116	1.022	1.218	0.014	1.276	0.987	1.650	0.063	1.177	0.683	1.827	0.660
*Annual high complexity case volume*																
< 25 cases	Reference				Reference				Reference				Reference			
25–50 cases	1.147	0.976	1.349	0.096	1.098	0.909	1.328	0.332	0.708	0.385	1.301	0.266	0.996	0.379	2.617	0.993
> 50 cases	1.306	1.175	1.452	0.001	1.196	1.057	1.354	0.005	1.206	0.836	1.741	0.317	0.680	0.358	1.289	0.237

Exp(B) for Cost and LOS represents the estimated ratio relative to the reference group, and exp(B) for readmission and mortality represents the odds ratio

### Reporting status and center volume

In analysis controlled for age, sex, and RACHS-1 severity, the STS reporting facilities were associated with an increased LOS (estimated means 16.3 and 14.6). Among STS reporting facilities, centers with a high volume of complex procedures demonstrated the same increase in length of stay (estimated means 16.2 and 13.5) with an accompanying increase in cost (estimated means $359,859 and $275,490). Readmission rates and inpatient mortality were not significantly different between reporting status and center volume (Table 3).

### Hazard ratios

**Fig. 1:**
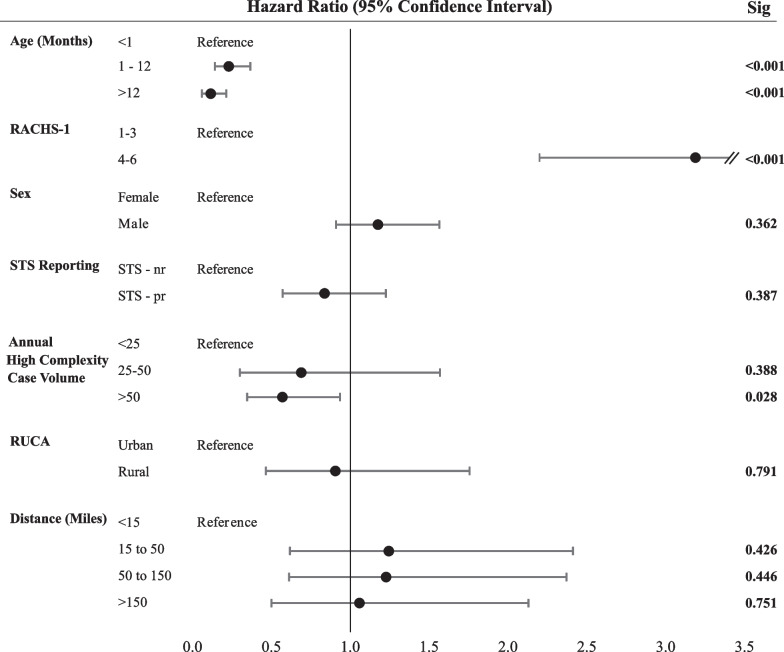
Hazard ratios (HR) for patient survival over a one-year period. Comparison of outcomes by sex were controlled for by age and RACHS-1 score. Remaining variable models included age, risk score, and sex. RUCA, Rural–Urban Commuting Area. RACHS-1, Risk Adjustment for Congenital Heart Surgery

RACHS-1 and age at first surgery demonstrated independent, significant hazard ratios for one-year mortality. Mortality was more than three times higher among patients with RACHS-1 scales 4–6 (HR 3.197, *p* < 0.001) and significantly lower among patients age > 12 months compared with < 1 month (HR 0.124, *p* < 0.001) (Fig. 1). Hazard ratios did not differ by sex, when controlling for RACHS-1 and age. Distance to care and rural origins were not associated with one-year mortality. Similarly, STS reporting status was not associated with one-year mortality. Among centers with available volume data, there is a significant trend of decreasing mortality with increasing volume (HR 0.579, *p* = 0.028).

## Discussion

Mortality among children with CHD in the first year of life is estimated at 37.8 per 100,000 [24]. Associated morbidity ranges widely from almost no-impairment to severe disability. As such, outcome improvement strategies are a major public health objective as stated by the American Heart Association and the Centers for Disease Control [25, 26]. Ongoing discussion often contrasts the proliferation of CHD centers with evidence for the regionalization of cardiac services.

The results of this study reinforce those of prior studies; increasing distance to a congenital heart disease center does not adversely affect mortality [27, 28]. Living farther from their primary treatment center, starting at > 150 miles, is associated with increased cost of admission. However, this association is without increased LOS or readmission rate.

Our additional analysis of a family’s rural origin allowed comparison of outcomes with consideration of local population density and distance to an urban core. Rural communities often lack easy access to complex, tertiary care. CHD care is no exception, often requiring long distance or even interstate travel. Complex regional insurance networks, varying coverage, and local population traits serve to confound comparisons. The Military Health System supports all DoD beneficiaries with direct and purchased care, including medical evacuation and family transportation whenever indicated. For this diverse, universally insured population, originating from a rural town or district conferred no mortality risk or measure of morbidity.

Importantly, these results reflect outcomes in families with regular and reliable access to prenatal care. We can assume that for more time dependent, critical CHD lesions, families were transferred as clinically appropriate to well-equipped surgical centers [29]. Alternatively, the effect of rare, severe, and unknown defects was too small to be represented in this retrospective review of patients from birth to ten years of age. To our knowledge, this study provides the first comparison of outcomes for CHD based on the STS-reporting status of the accepting institution. Our analysis found no mortality benefit to STS-reporting centers. There was evidence of increased LOS. Among STS-centers, we compared outcomes between high volume centers (> 50 high-complexity cases annually) and low volume centers (< 50 and < 25 high-complexity cases annually). While no difference in inpatient mortality, there was a significant trend of decreasing one year mortality with increasing volume.

While high volume STS-reporting centers were associated with increased cost and LOS, they were highly sought after. Families traveled farther for these centers, particularly high-volume centers. Indeed, the factors best correlated with increased distance to care were STS-reporting status, center volume, and the patient’s RACHS-1 score. This pattern fits well with an assumption that families and their advising providers direct patients with increasingly complex pathology to high volume, well experienced centers. While we controlled for RACHS-1 severity, we cannot account for these preferential referral patterns.

### Generalizability

Some strengths of our study also limit its generalizability. Our population is universally insured, employed, often with a higher education level than the general US population, and may not reflect the experience and outcomes of other families originating from rural or small-town communities. Active duty families may lack the same environmental exposures or local genetic variations that could account for observed differences in CHD rates for rural populations [30]. As discussed above, a limitation of this dataset is that it also lacks some factors used in other studies to estimate or explain excess mortality, such as race, ethnicity, or socioeconomic status [31, 32].

### Limitations

As is standard for billing database analyses, no detailed chart information is available to assess timing of referral or medical decision making. Due to this chosen methodology, there is no information on patients with CHD who did not receive surgical intervention. Similarly, we are unable to assess if the decision for catheter-based intervention may have affected outcomes.

An important limitation of our study includes the effect of prenatal diagnosis and EFMP. Indeed, a prenatal diagnosis and family compliance with the EFMP program should preclude movement of complex care patients to a distant or rural duty station. Families stationed remotely still have access to regular prenatal care, theoretically allowing time for transfer and delivery at a well-equipped medical center. Previous studies have demonstrated a mortality benefit for prenatal diagnosis of certain critical CHD [29]. Our method of analysis using available billing data precluded an assessment of fetal diagnosis and its relation to where families lived at the time of surgery. No study has prospectively evaluated the effect of prenatal diagnosis and the actual relocation of families from distant locations, but we would theorize the effect on our population to be positive.

In previous studies, differences in mortality were described with hospital-specific, standardized mortality rates, such as in Chan and colleagues or by controlling for prematurity and other comorbidities, such as non-cardiac malformations or genetic syndromes [12, 33]. Such granular analysis with a more complete sampling of surgical center volumes at STS-nr may have elucidated more differences between low and high-volume centers in our population. Additional covariables not available for our analysis, such as the time of diagnosis, institute complication rate, and comparison between specific surgical procedures may have affected outcomes. Facility volume and reporting status were not available for the exact time frame for which patient data are reported, although we expect that changes over time were minimal. Finally, the analysis presented is retrospective. Following studies should be prospective, with focus on outcomes for patients with high-risk procedures at selected regional centers of excellence.

## Conclusions

Overall, these data support regionalization of CHD surgical care. A family’s distance to care and increasing rurality did not adversely impact patient mortality. While distance to care increased cost, other correlates of morbidity, including readmission rate or LOS, were unaffected. Centers with a high annual volume of high-risk surgeries demonstrated decreased mortality, despite an increased cost and length of stay. Future studies will aim to follow such critical patients through both initial diagnosis to surgery and from surgery to long term outcomes as it relates to residence.

## Data Availability

The dataset generated and analyzed during the current study is not publicly available due to data use restrictions in place by the United States Department of Defense. To discuss access and data availability, please contact both Dr. Ryan Flanagan at ryan.p.flanagan2.mil@health.mil and Dr. Spencer Millen at spencer.m.millen.mil@health.mil.

## Abbreviations

CHD: Congenital heart disease
DEERS: Defense enrollment eligibility reporting system
DoD: U.S. Department of Defense
EFMP: Exceptional family members program
LOS: Length of stay
RACHS-1: Risk adjustment for congenital heart surgery
RUCA: Rural–urban commuting area codes
STS: Society of thoracic Surgery
STS-pr: Society for thoracic surgery database, reporting
STS-nr: Society for thoracic surgery database, non-reporting
